# Effects of sodium bicarbonate on cell growth, lipid accumulation, and morphology of *Chlorella vulgaris*

**DOI:** 10.1186/s12934-018-0953-4

**Published:** 2018-07-09

**Authors:** Jingya Li, Changhao Li, Christopher Q. Lan, Dankui Liao

**Affiliations:** 1 Guangxi Key Laboratory of Petrochemical Resource Processing and Process Intensification Technology, School of Chemistry & Chemical Engineering, Guangxi University, Nanning, 530004 China; 2 Department of Chemical and Biological Engineering, University of Ottawa, Ottawa, ON K1N 6N5 Canada

**Keywords:** Cell growth, Cell morphology, DIC effects, *Chlorella vulgaris*

## Abstract

**Background:**

Low concentration NaHCO_3_ (ca. 12 mM) had been demonstrated to be an excellent carbon source for industrially important green alga *Chlorella vulgaris* and high concentration NaHCO_3_ (e.g. 160 mM) had been shown to be capable of controlling protozoa and stimulating lipid accumulation of another green alga, i.e., *Neochloris oleoabundans*. Furthermore, little was known about the mechanisms of the effects of NaHCO_3_ on microalgae. Thorough studies on the effects of high NaHCO_3_ on *C. vulgaris* and their mechanisms were therefore warranted.

**Methods:**

We systematically compared the cell growth, lipid production, and cell morphology of the industrially important *C. vulgaris* in 160 mM NaHCO_3_ or 160 mM NaCl media at different pH levels. These data allowed us to analyze the effects of total dissolved inorganic carbon (DIC) and individual DIC species on *C. vulgaris*. Cell growth of *C. vulgaris* at a range of concentrations at 160 mM or lower was also studied.

**Results:**

Cellular lipid cell content of 494 mg g^−1^ and lipid productivity of 44.5 mg L^−1^ day^−1^ were obtained at 160 mM NaHCO_3_ and pH 9.5. High concentration NaHCO_3_ (e.g. 160 mM) was inhibitive to cell growth but stimulating to lipid accumulation and caused unicellular *C. vulgaris* to transfer to colonial cells. Increasing pH in the range of 7.5–9.5 caused increasing inhibition to cell growth in 160 mM NaCl. Whereas the optimal pH for cell growth was 8.5 for 160 mM NaHCO_3_ cultures. Comparative experiments with 0–160 mM NaHCO_3_ indicate that 10 mM was the optimal concentration and increasing NaHCO_3_ from 10 to 160 mM caused increasing inhibition to cell growth.

**Conclusions:**

High concentration DIC was inhibitor to cell growth but stimulator to lipid accumulation of *C. vulgaris*. It caused unicellular *C. vulgaris* to transform to colonial cells. Results suggest that high concentration of a particular DIC species, i.e., dCO_2_, was the primary stress responsible for cell growth inhibition. Where CO_3_^2−^ was likely the DIC species responsible for lipid stimulation of *C. vulgaris*. Furthermore, we propose that the colony formation at high DIC conditions was employed by *C. vulgaris* to mitigate the stress by minimizing cell exposure to unfavorable environment.

## Background

Freshwater green alga *Chlorella vulgaris* is an industrially important microalgal species, which has an annual global production of approximately 2000 tons [1]. Its biomass is typically composed of 40% proteins, 25% lipids, 20% carbohydrates, 5% fiber, 10% minerals and a variety of different vitamins [2], lending it great potential as a source of proteins [3] and other nutrients for humans and animals [4, 5]. The lipid content of *C. vulgaris* could be increased significantly to the range of 50–70% when lipid stimulus is included, making it a promising feedstock for biodiesel production [6]. The high cost of cultivation and harvest, however, has been one of the major concerns in industrial farming of *C. vulgaris* and other microalgae, which is of particularly relevance when production of microalgal biofuel is attempted [7]. In fact, the prohibitive costs and energy consumption are two major factors impeding commercial algal fuel production.

NaHCO_3_ was found a good carbon source for *C. vulgaris* strains with extracellular carbonic anhydrase (CA) at cell surface, which could serve as transporters of HCO_3_^−^ under low dissolved CO_2_ (dCO_2_) conditions [8]. As an illustrative example, a *C. vulgaris* strain isolated from sewerage treatment plant was grown in the presence of 3.0 mM (250 mg L^−1^) to 12 mM (1000 mg L^−1^) sodium bicarbonate as carbon source with the specific growth rate increased continuously in the range with no significant impacts on cellular lipid content [9]. It was found in another research that *C. vulgaris* could remove CO_2_ much more efficiently when NaHCO_3_ instead of gas CO_2_ was used as the carbon source [10]. It is also worth mentioning that NaHCO_3_ is much easier to handle than gaseous CO_2_ in storage, transportation, and processing and is therefore a cost-effective alternative carbon source when applicable. These studies, nonetheless, were carried out at low NaHCO_3_ concentrations (12 mM or less).

Our previous studies with another freshwater green alga *Neochloris oleoabundans* indicate that NaHCO_3_ at relatively high concentration (80–160 mM) could stimulate lipid production [11] but this has not been reported with *C. vulgaris*. Furthermore, previous studies in our lab demonstrated that the addition of 160 mM sodium bicarbonate in combination with fine-tuned culture pH (8.5–9.5) is capable of controlling protozoa to allow safe cultivation of freshwater microalga *N. oleoabundans* [12]. These findings opened a window for safe cultivation of freshwater microalgae in open systems, which could potentially be devastated by protozoa [13]. These studies, nonetheless, focused on the inhibition of protozoa by NaHCO_3_ rather than the effects of high NaHCO_3_ on microalgal cells.

In this study, we systematically compared the cell growth, lipid production, and cell morphology of the industrially important *C. vulgaris* in 160 mM NaHCO_3_ or 160 mM NaCl media at different pH levels. These data allowed us to analyze the effects of total dissolved inorganic carbon (DIC) and individual DIC species on *C. vulgaris*. Cell growth of *C. vulgaris* at a range of concentrations at 160 mM or lower was also studied, which revealed that NaHCO_3_ was beneficial at low concentration as carbon source but inhibitive at high concentration. Transformation of unicellular *C. vulgaris* cells to colonial cells was observed under the stress of high concentration NaHCO_3_. These colonial cells were sensitive to mild shear stress, confirming the hypothesis that colony formation was a mechanism for cells to resist the stress of high DIC concentration by minimizing exposure to stressful environment.

## Methods

### Strain and medium

*Chlorella vulgaris* UTEX 2714 was purchased from the algae culture collection at the University of Texas in Austin. The modified Bristol medium (MBM) was used in this study, which was composed of (per liter and analytical grades) 0.35 g NaNO_3_, 0.138 g K_2_HPO_4_, 0.0823 g MgSO_4_, 0.025 g CaCl_2_, 0.322 g KH_2_PO_4_, 0.025 g NaCl, 0.0068 g FeCl_3_ and the A_5_ solution, which was comprised of the following components (per liter): 2.86 mg H_3_BO_3_, 1.81 mg MnCl_2_·4H_2_O, 0.22 mg ZnSO_4_·7H_2_O, 0.079 mg CuSO_4_·5H_2_O, 0.039 mg (NH_4_)_6_Mo_7_O_24_·4H_2_O. NaHCO_3_, KHCO_3_, NaCl or KCl was added at varied concentrations as specified in the text when applicable.

### Cultivation of microalgae

Cultivations of microalgae were carried out at 27 °C under a continuous illumination of approximately 360 µmol m^−2^s^−1^.

Pre-cultures were prepared in flasks containing 135 mL fresh MBM medium. The medium pH was adjusted with HCl and NaOH to 7.5 before autoclaving. The autoclaved flasks were cooled to room temperature, inoculated with 15 mL inoculum, and then incubated in a plant growth chamber (NO. LI15, Sheldon Manufacture, Inc. USA) for approximately 10 days. The chamber was supplied with an air stream enriched with 5% CO_2_. The flasks were hand-shaken three times every day to enhance gas exchange and mixing.

Cultivation experiments were carried out in 1-L cultivation bottles, which were located in a custom-designed illumination box, or 3-L photobioreactors (PBRs), which were converted from BioFlo 110 Bioreactors (New Brunswick Scientific GMI Inc., Canada) by installing a set of 12 white fluorescent lights surrounding each bioreactor. The working volumes were 0.8 and 2 L for the bottles and PBRs, respectively.

For microalga cultivation in 1-L bottles, cultivation bottles containing 720 mL medium was adjusted to the pH as specified in the text and then autoclaved. The sterilized bottles were connected to CO_2_-enriched air stream (containing 5% CO_2_) at a flow rate of 0.5 vvm. Pre-culture of 80 mL was added to each bottle under sterile condition after the autoclaved media was cooled to room temperature. The air stream was passed through a bottle filled with distilled water for miniaturization before being filtered through a 0.27 μm membrane disc filter at the inlet of the cultivation bottle. A magnetic stir for each bottle was used to provide agitation and enhance gas exchange. Samples were taken for measurement on a daily basis.

For cultivation experiments in PBRs, PBRs were autoclaved and then loaded with 2.0 L of medium each before being connected to filtered air at 0.5 vvm and turning on agitation and cooling water. Appropriate volume of pre-culture was inoculated to the fresh medium to bring an initial biomass concentration up to about 0.1 g L^−1^. Culture pH was controlled at the set values by adjusting flowrate of CO_2_ using the gas mixer linked to the control panel of the PBR. Illumination was provided by 12 fluorescence lamps (Philips Plant & Aquarium T18/15, Philips Electronics Ltd. ON, Canada) and the culture was maintained at 27 °C with the cooling jacket. Agitation speed was kept at 100 rpm or otherwise specified in the text. Culture samples were taken for analysis daily.

### Analytical methods

#### Cell morphology, cell/colony size and biomass concentration

Biomass concentration of microalgal cultures were determined by measuring the optical density of samples at a wavelength of 720 nm (OD_720_) using a spectrophotometer (GENESYS 10 uv, Thermo Electron Co., USA). Samples were diluted to ensure the measured OD_720_ values were in the range of 0.2–0.4 if applicable. Biomass concentration was determined by multiplying the OD_720_ values with a pre-determined conversion factor determined as the slope of the linear portion of a calibration curve.

#### Morphology and size of microalgae

The morphology of microalgae was observed under a phase contrast microscope (Infinity II BX40, Olympus, Canada) at 600× magnification. The sizes of cells and colonies were determined by analyzing the micrographs of typical samples using ImageJ.

#### Lipids

Biomass was harvested from the culture suspension at the end of batch cultivation for lipid analysis. Lipids were extracted in Soxhlet extractor by ethyl ether from approximately 1 g dry biomass after drying in an oven at 105 °C until constant weight. After extraction for 6–8 h in Soxhlet extractor, the residue was dried at 80 °C for 2 h and weighed after cooling to room temperature. Cell lipid content (LC) is calculated using Eq. 1.

1 $$ {\text{LC}} = \frac{{m_{0} - m}}{{m_{0} }} $$

where *m*_0_ and *m* are the mass of the dry biomass sample before and after Soxhlet extraction, respectively.

Neglecting the small amount of lipids contained in the initial biomass, lipid productivity (LP, mg L^−1^ day^−1^) was calculated using Eq. 2:

2 $$ {\text{LP}} = \frac{{   {\text{LC}} \times {\text{DCW}}}}{\text{Time}} $$

where DCW is biomass concentration of a culture in dry cell weight (g L^−1^) and time is the cultivation time in days.

All measurements were done in triplicates.

## Results and discussion

### *Chlorella vulgaris* growth in 160 mM NaCl or NaHCO_3_ at different pH

The effects of 160 mM NaHCO_3_ on cell growth, lipid production, and morphology of *C. vulgaris* were studied in comparison to that of 160 mM NaCl for three reasons: (1) 160 mM NaCl has an osmotic pressure similar to that of 160 mM NaHCO_3_ in the pH range of 7.5–9.5; (2) NaCl concentration (i.e., salinity) has been commonly studied as a factor affecting both cell growth and lipid production of microalgae, including *C. vulgaris*; and (3) similar to 160 mM NaHCO_3_, 160 mM NaCl was demonstrated to be able to control protozoa in a recent study as well [14] and could be used to replace NaHCO_3_ for that purpose if it is more beneficial in terms of cell growth, lipid production, or other applications.

**Fig. 1:**
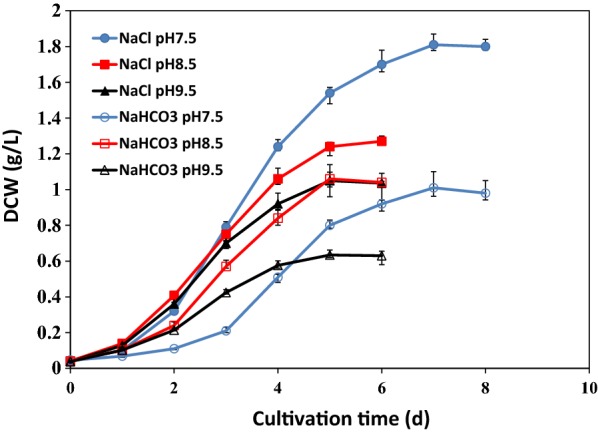
Cell growth of *C. vulgaris* in media containing 160 mM NaHCO_3_ or NaCl at pH 7.5, 8.5 or 9.5. Experiments were carried out in PBR and reported are mean values ± standard errors of triplets

Figure 1 shows that the cell growth of *C. vulgaris* was better in NaCl media than in NaHCO_3_ media at all three pH levels. For instance, the maximum biomass concentration in NaCl cultures was 1.8 g DCW L^−1^ at pH 7.5, almost doubling that in NaHCO_3_ cultures at the same pH (1.1 g DCW L^−1^). As discussed in “Effects of NaHCO_3_ concentration on cell growth of *C. vulgaris*” section, the cell growth in the control (i.e., MBM) was only slightly higher than that in 160 mM NaHCO_3_ due to the interplay of NaHCO_3_ stress and the increased supply of carbon source. Therefore, it is reasonable to conclude that adding 160 mM NaCl to media could stimulate the cell growth of *C. vulgaris*. These results are compatible with that of Luangpipat and Chisti [15], who compared the growth and lipid production of *C. vulgaris* in media of different salinities formulated with freshwater, seawater, or 50% seawater. The 50% seawater medium, which contained approximately 300 mM NaCl, was the best for both cell growth and lipid production. These results indicate that, although extremely high salinity such as that of the seawater is inhibitive to cell growth, addition of moderate amount of NaCl, i.e., 160 or 300 mM, could stimulate cell growth.

The osmotic pressure of 160 mM NaCl and 160 mM NaHCO_3_ are similar to each other because, as will be discussed in “Effects of pH and individual DIC species” section, HCO_3_^−^ was the predominant DIC species at all the three pH levels tested in this study. Therefore, the major difference between 160 mM NaHCO_3_ and 160 mM NaCl cultures at the same pH was that the total DIC of the former was much (approximately 160 mM) higher than in the latter, which relied solely on aeration for dCO_2_ supply. Therefore, the data shown in Fig. 1 provide a basis for comparing the effects of total DIC at similar osmotic pressure and the same pH.

As shown in Fig. 1, cell growth decreased continually with the increase of pH in the range of pH 7.5 to pH 9.5 in 160 mM NaCl cultures, suggesting pH higher than neutrality was inhibitive to cell growth and the adverse effect increased with pH. However, the optimum pH shifted to 8.5 when *C. vulgaris* was cultivated in MBM containing 160 mM NaHCO_3_, which is in accordance with the results of our previous studies with another freshwater green alga *N. oleoabundans* [12]. The different impacts of pH on cell growth of *C. vulgaris* in 160 mM NaCl and that in 160 mM NaHCO_3_ are of great interest, which will be examined in “Effects of pH and individual DIC species” section.

### Lipid accumulation of *C. vulgaris* in 160 mM NaCl or NaHCO_3_ at different pH

**Fig. 2:**
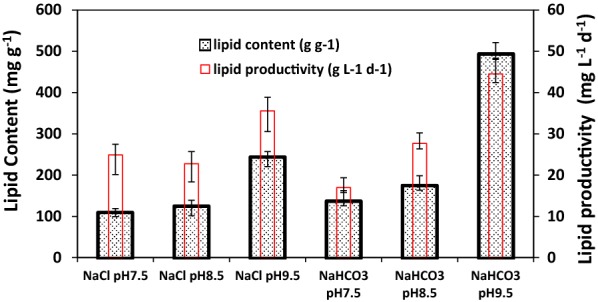
Lipid production of *C. vulgaris* in media containing 160 mM NaHCO_3_ or 160 mM NaCl at pH 7.5, 8.5 or 9.5. Cells were harvested for lipid measurement at the end of cultivation as shown in Fig. 2. Reported are the mean values ± standard errors of triplets

Biomass was harvested at the end of cultivation to measure lipid content (LC) and lipid productivity (LP). The results are shown in Fig. 2.

When the effect of pH in cultures containing the same salt, i.e., NaCl or NaHCO_3_, is compared, the same trend was observed, i.e., the LC of *C. vulgaris* increased slightly when culture pH increased from 7.5 to 8.5 and significantly when pH further increased to 9.5 in both NaCl and NaHCO_3_ cultures. The lipid stimulating effect of high pH level are consistent with that of Kwak et al. [16], who reported that the LC of *C. vulgaris* cultivated in TP medium with 200 mM NaCl increased significantly from 17.99 ± 0.17% at neutral pH to 26.84 ± 0.25% at pH 10.0.

Comparing the LC of *C. vulgaris* cells from NaCl and NaHCO_3_ containing cultures at the same pH levels, it is clear that NaHCO_3_ is a stronger stimulus for lipid accumulation than NaCl since the LC of NaHCO_3_ cultures were higher than NaCl cultures at all three pH levels. At pH 9.5, the LC of cells from NaHCO_3_ cultures was 494.0 mg g^−1^, which was more than doubling that of the NaCl cultures at the same pH (244.8 mg g^−1^). The LC of cells from NaHCO_3_ culture at pH 9.5 was high enough to compensate the relatively low biomass concentration when compared to NaCl cultures at pH 9.5 and the LP of the former (44.5 mg L^−1^ days^−1^) was significantly higher than that of the latter (33.56 mg L^−1^ days^−1^).

The inhibition of high pH to cell growth as shown in NaCl cultures (Fig. 1) could be explained by the two factors that reinforce each other when pH increases from the more favorable pH 7.5 to the unfavorable pH 9.5. Firstly, cells need to constantly pump protons (H^+^) against its concentration gradient from the basic extracellular environment, e.g., [H^+^] ≅ 3.33 × 10^−10^ M for cultures at pH 9.5, to cytoplasm to maintain the intracellular environment at the pH optimum, which is likely to be near the neutrality (i.e., [H^+^] ≅ 10^−7^ M). This is an energy consuming process, which would biogenetically disadvantage cells in high pH cultures than cells at the more favorable pH (for instance, pH 7.5 in this study). In other word, more bioenergy needs to be consumed for cell maintenance when culture pH is away from the optimum and the further away the culture pH goes, the more bioenergy to be expended for cell maintenance, reducing the bioenergy that could be otherwise directed to cell growth. Secondly, the ratio of dCO_2_ among total DIC decreases exponentially with the increase of pH and would approach zero at pH 9.5 (pK_1_ = 6.381, pK_2_ = 10.337 for carbonate equilibrium). As a result, *C. vulgaris* at high pH would increasingly rely on the energy consuming CCM for assimilating HCO_3_^−^ as carbon source, which is bioenergetically disadvantaged in comparison to assimilation of dCO_2_ by molecular diffusion across the cytoplasmic membrane. However, the effects of pH is more complicate due to the involvement of DIC and will be discussed in detail in “Effects of pH and individual DIC species” section.

### Formation of colonies by *C. vulgaris* under stress of high NaHCO_3_

**Fig. 3:**
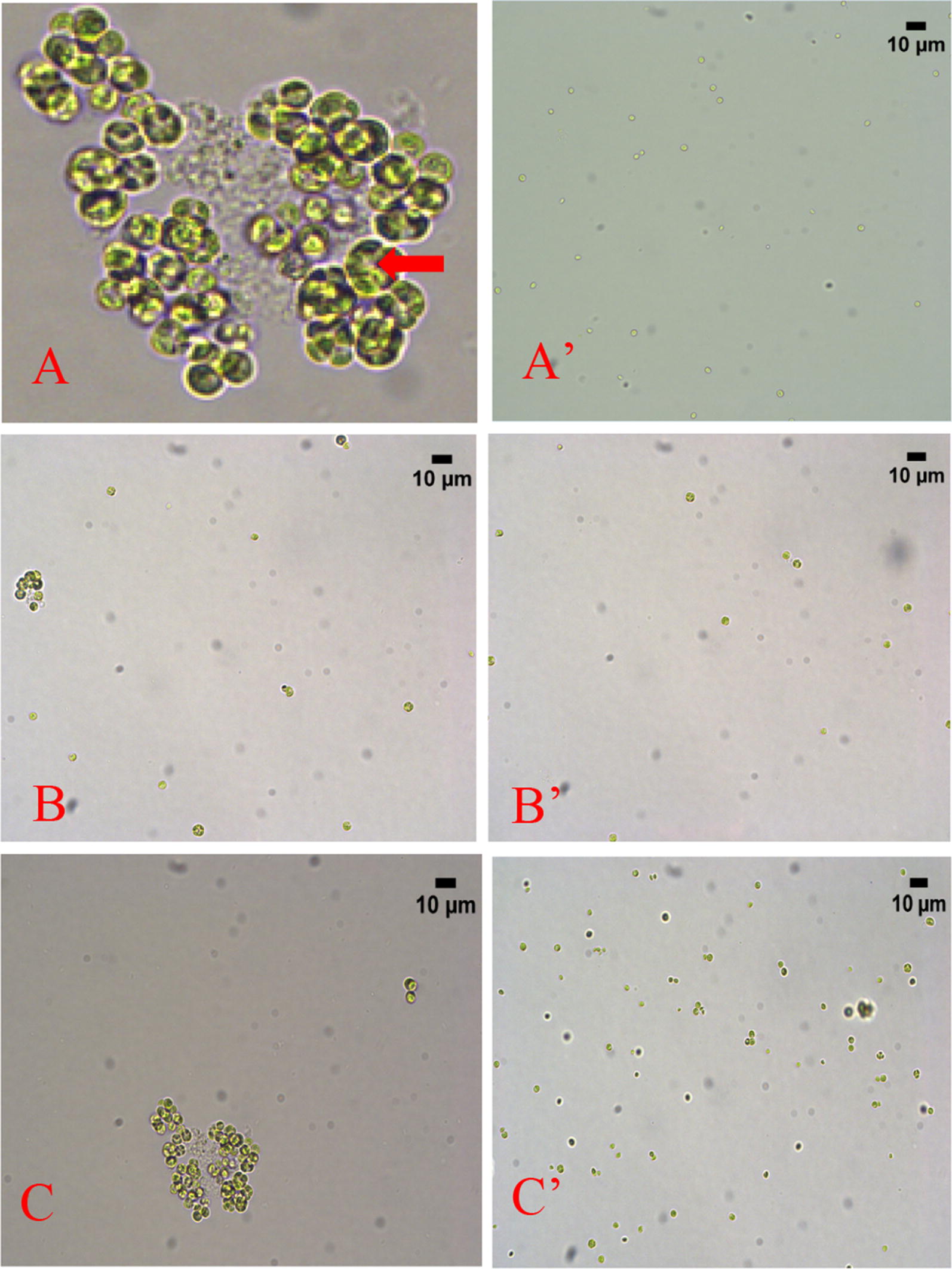
Colonies and clusters of *C. vulgaris* in Day-4 160 mM NaHCO_3_ culture (**A**); unicellular cells in pre-culture/cell size 1.85 ± 0.41 μm (**A’**), Day 2 160 mM NaCl culture/cell size 2.84 ± 0.70 (**B’**) and Day-2 160 mM NaHCO_3_ culture/cell size 3.21 ± 0.73 μm (**B**); and colonies in Day-4 160 mM NaHCO_3_ culture/colony size 4.24 ± 1.09 μm (**C**), and predominantly unicellular cells in Day 4 160 mM NaCl culture/cell size 2.23 ± 0.35 μm (**C’**). All micrographs were taken at 600 magnification except Graph A, which is the enlargement of part of Graph C. The red arrow in Graph A points to a colony in a cluster. Samples were diluted and then vortexed before microscopic examination for clarity when needed. Clusters as shown in Graph A were predominant in 160 mM NaHCO_3_ cultures on Day 4 and thereafter

Figure 3 compares the morphologies of cells in 160 mM NaHCO_3_ cultures (A, B, and C), pre-cultures (A′) and 160 mM NaCl cultures (B′ and C′) on different days of cultivation.

As shown in Fig. 3, cells in pre-cultures, which were grown with MBM (with no additional NaHCO_3_ or NaCl), were all unicellular. Cells remained unicellular in both 160 mM NaCl and 160 mM NaHCO_3_ cultures after one day of incubation (Day 2). However, colonies appeared in the third day and become dominant from the fourth day of cultivation (Day 4) in the NaHCO_3_ cultures. In fact, as highlighted in Graph A, in 160 mM NaHCO_3_ cultures it was common for several colonies to form loose clusters and the colonies in the same cluster appeared to be secondary colonies derived from cells of a single primary colony through second-generation divisions. These colonies and colony clusters were dominant in 160 mM NaHCO_3_ from Day 4 and thereafter. On the other hand, some small colony-like cells appeared in Day 4 160 mM NaCl cultures and thereafter but most cells remained unicellular.

Cells grown in the pre-culture, which was prepared with MBM at pH 7.5, had a mean cell size of 1.85 ± 0.41 μm. However, the sizes of the unicellular cells in Day-2 160 mM NaCl cultures and Day-2 NaHCO_3_ cultures were 2.84 ± 0.70 and 3.21 ± 0.73 μm, respectively. Furthermore, the size of cells in Day-4 160 mM cultures was 2.23 ± 0.35 μm and that of the colonies in Day-4 160 mM NaHCO_3_ cultures was 4.24 ± 1.09 μm. In other words, adding 160 mM NaCl or NaHCO_3_ would cause cells to become larger than that in MBM cultures and the cell size in 160 mM NaHCO_3_ cultures were larger than that in 160 mM NaCl cultures.

It was reported that the presence of protist predators, *Tetrahymena* *thermophile* [17] and *Ochromonas vallescia* [18], or the supernatants taken from predator cultures were able to promote colony formation of *C. vulgaris*. It has been also reported that predator exoproducts promoted colony formation in other green algae, including *Microcystis* [19]*, Scenedesmus* [19, 20]*, Phaeocystis* [21] and *Chlamydomonas* [22]*, Microcystis aeruginosa* [23, 24]. In these studies, it was argued that colony formation was a defensive mechanism of microalgae and other unicellular microorganisms against predators [25] and detecting predator exoproducts (e.g. waste products, pheromones) could be a quick and reliable way for microalgae to respond to the presence of specific predators, before the predators actually become a direct threat [26]. Our results indicate that microalgae could also form colonies in response to a stress derived from the presence of relatively high concentration of inorganic chemicals such NaHCO_3_, possibly to employ multicellularity to minimize the exposure of cells to the stress of high NaHCO_3_. This is the first time colony formation in response to the existence of inorganic chemicals has been reported with microalgae to our best knowledge.

### Effects of sheer stress on *C. vulgaris* in cultures containing NaHCO_3_ at different pH

Table 1 shows the influence of sheer stress (100 and 200 rpm) on cell growth and lipid accumulation in cultures containing 160 mM NaHCO_3_ with pH controlled at 7.5 and 9.5, respectively. The increase of agitation from 100 to 200 rpm caused reduction of biomass concentration, maximum specific growth, LC, and LP of *C. vulgaris* at both pH 7.5 and pH 9.5 and cells at pH 9.5 were more severely affected.

**Table 1:** Cell growth and lipid accumulation of *C. vulgaris* in cultures containing 160 mM NaHCO_3_ at varied agitation and pH

	pH 7.5		Δ%	pH 9.5		Δ%
	100 rpm	200 rpm		100 rpm	200 rpm	
DCW_max_ (g L^−1^)	1.01 ± 0.11	0.8 ± 0.072	− 20.8	0.63 ± 0.086	0.52 ± 0.043	− 17.5
μ_m_ (day^−1^)	0.49 ± 0.062	0.42 ± 0.038	− 14.3	0.31 ± 0.0412	0.25 ± 0.0215	− 19.4
Lipid content (mg g^−1^)	138 ± 18.43	112 ± 14.58	− 18.8	494 ± 20.42	327 ± 14.65	− 33.8
Lipid productivity (mg L^−1^ day^−1^)	16.98 ± 2.98	12.8 ± 2.06	− 24.6	44.5 ± 2.91	24.3 ± 1.86	− 45.4

While some microalgae belonging to diatoms and red algae [27] have been shown to be sensitive and some others such as dinoflagellates [28, 29] extremely sensitive to shear stress, *C. vulgaris* has shown the ability to tolerate high shear stress. *C. vulgaris* has a unilamellar cell wall of 17–21 nm, contains a rigid microfibrillar layer composed of glucosamine [30] and, in the form of unicellular cells, have a cell size of 5–10 µm in diameter [3]. This unique structure lends it high shear tolerance in regular media, which has been well demonstrated in a variety of different settings including stirred-tank [31], bubble column [32], and tubular PBR with centrifugal pump for culture recirculation [33].

The increased sensitivity of *C. vulgaris* to sheer stress in cultures containing 160 mM NaHCO_3_ is likely associated with the stress that caused the change of cell transformation from unicellular to colonial. It was reported that [34] filamentous microalgae or cyanobacteria are typically sensitive to shear stress but colonial microalgae in general benefits from mild agitation, hypothetically due to the breakup of colonies into individual cells that facilitates mass transfer. With reference to these findings, we hypothesize that even a mild increase of agitation from 100 to 200 rpm in media containing 160 mM NaHCO_3_ would make it more difficulty for cells to form protective colonies and therefore more exposed to the stress of high DIC. This hypothesis could also account for the more severe shear-sensitivity of *C. vulgaris* in 160 mM NaHCO_3_ cultures at pH 9.5 than at 7.5 because the former, which is a unfavorable pH, is more stressful than the latter.

### Effects of pH and individual DIC species

At constant temperature, the following equilibria between different DIC species apply:

3 $$ CO_{2} \left( {aq} \right) + H_{2} O \leftrightarrow H_{2} CO_{3} \mathop \leftrightarrow \limits^{{K_{1} }} H^{ + } + HCO_{3}^{ - } \mathop \leftrightarrow \limits^{{K_{2} }} 2H^{ + } + CO_{3}^{2 - } $$

where dCO_2_ = [CO_2_(*aq*)] = [H_2_CO_3_] and

4 $$ K_{1} = \frac{{\left[ {H^{ + } } \right] \times \left[ {HCO_{3}^{ - } } \right]}}{{[H_{2} CO_{3} ]}} = \frac{{\left[ {H^{ + } } \right] \times \left[ {HCO_{3}^{ - } } \right]}}{{dCO_{2} }} $$

5 $$ K_{2} = \frac{{\left[ {H^{ + } } \right] \times \left[ {CO_{3}^{2 - } } \right]}}{{\left[ {HCO_{3}^{ - } } \right]}}. $$

The total concentration of dissolved inorganic carbon ([C_T_]) in the culture can be expressed as:

6 $$ \left[ {DIC} \right] = dCO_{2} + \left[ {HCO_{3}^{ - } } \right] + \left[ {CO_{3}^{2 - } } \right]. $$

Combining Eqs. 3–6, and reorganizing gives:

7 $$ F_{{dCO_{2} }} = \frac{{dCO_{2} }}{{\left[ {DIC} \right]}} = \frac{1}{{1 + \frac{{K_{1} }}{{\left[ {H^{ + } } \right]}} + \frac{{K_{1} K_{2} }}{{[H^{ + } ]^{2} }}}} $$

8 $$ F_{{HCO_{3}^{ - } }} = \frac{{\left[ {HCO_{3}^{ - } } \right]}}{{\left[ {DIC} \right]}} = \frac{1}{{\frac{{\left[ {H^{ + } } \right]}}{{K_{1} }} + \frac{{K_{2} }}{{\left[ {H^{ + } } \right]}} + 1}} $$

9 $$ F_{{CO_{3}^{2 - } }} = \frac{{\left[ {CO_{3}^{2 - } } \right]}}{{\left[ {DIC} \right]}} = \frac{1}{{\frac{{[H^{ + } ]^{2} }}{{K_{1} K_{2} }} + \frac{{\left[ {H^{ + } } \right]}}{{K_{2} }} + 1}} $$

where that *pK*_1_ = 6.381 and *pK*_2_ = 10.337 at 25 oC and *pH *= − log[H^+^]. Accordingly, dCO_2_, [HCO_3_^−^] and [CO_3_^2−^] at pH 7.5, 8.5, and 9.5 could be calculated for 160 mM NaHCO_3_ cultures assuming the contribution of aeration to DIC was negligible and the culture properties are identical to pure water. The results are listed in Table 2. For the purpose of comparison, the pCO_2_ of gas phase required to achieve the same dCO_2_ in pure water if such a gas stream were used for aeration at 25 °C, assuming Henry’s law, i.e., Eq. 10, was applicable. Results are presented in Table 2 as well.

10 $$ pCO_{2} = K_{CO2} \times dCO_{2} $$

where pCO_2_ is the partial pressure of CO_2_ in the gas (atm), and $K_{{{\text{CO}}_{ 2} }}$ is the Henry’s constant of CO_2_ (i.e., 29.41 L atm mol^−1^).

**Table 2:** Concentration of individual DIC species, cell growth, and lipid production of *C. vulgaris* in 160 mM NaHCO_3_ cultures at varied pH

	dCO_2_	pCO_2_^a^ (atm)	[HCO_3_^−^]	[CO_3_^2−^]	DCW_m_ (g L^−1^)	LC (mg g^−1^)	LC^b^ (mg g^−1^)
pH 7.5	11.29	0.332	148.49	0.22	1.00	138.1	110.6
pH 8.5	1.19	0.035	156.53	2.28	1.20	175.9	126.0
pH 9.5	0.11	0.0003	139.58	20.32	0.60	494	244.81

^a^Partial CO_2_ pressure in aeration stream assuming the same dCO_2_ was obtained by bubbling pure water at 25 °C using Henry’s law is applicable (K_H_ = 29.41 atm mol^−1^)

^b^LC of 160 mM NaCl cultures at different pH levels

As shown in Table 2, [HCO_3_^−^] was the dominant species at all three pH levels and its concentration varied only slightly between 139.9 and 156.5 mM in this pH range. Therefore, the vast difference in both cell growth and lipid production occurred at different pH levels in 160 mM NaHCO_3_ cultures were unlikely to be caused by the concentration change of this species. On the other hand, dCO_2_ decreased more than 100 times from 11.3 mM at pH 7.5 to 0.11 mM at pH 9.5 while [CO_3_^2−^] increased 94 times from 0.22 at pH to 20.32 mM at pH 9.5. It might be reasonable to argue that the concentration of species dCO_2_ dominates the impact of DIC on cell growth of *C. vulgaris* for the following reasons. First, dCO_2_ is the conventional carbon source for photoautotrophic growth of microalgae and dCO_2_ of 0.11 and 11.29 mM are equivalent to the dCO_2_ if pure water is brought to equilibrium with a gas phase CO_2_ content of 0.3 and 33.3%, respectively. While the former is limiting, the latter is inhibitive to the cell growth of most microalgal species. The dCO_2_ of 160 mM NaHCO_3_ cultures at pH 8.5 is 1.19 mM, corresponding to a pCO_2_ 0.035 atm (or 3.5% CO_2_ content) in gas, which is the level of dCO_2_ suitable for *C. vulgaris*.

Since [HCO_3_^−^] was abundant in 160 mM at all three pH levels, supply of carbon sources should have not been a problem. However, assimilating [HCO_3_^−^] through CCM using CA at cell surface as the [HCO_3_^−^] transporter is energy consuming and therefore bioenergetically disadvantaged. This could explain the reduced cell growth of *C. vulgaris* in both NaCl and NaHCO_3_ cultures when comparing cell growth at pH 9.5 with that that in the corresponding pH optima (i.e., pH 7.5 for NaCl cultures and pH 8.5 for NaHCO_3_ cultures).

The contribution of [CO_3_^2−^] to cell growth of *C. vulgaris* is probably secondary, if any. As an anion, [CO_3_^2−^] could not cross the cytoplasmic membrane by molecular diffusion as the neutral dissolved CO_2_ molecule does and there is no known active transportation mechanism for its assimilation as is the case of HCO_3_^−^ assimilation.

The data presented in Table 2 (and Fig. 2) indicate that the LC of cells in 160 mM NaHCO_3_ and 160 mM NaCl cultures had the same trend, i.e., increasing with pH in the range of 7.5–9.5 but the latter had higher LC levels at all three pH levels. We hypothesize that the increase of [CO_3_^2−^] with pH was the primary reason for the lipid stimulating effects of DIC due to the following reasons: (1) dCO_2_ approached zero at pH 9.5 as shown in Table 2 but high lipid production was reported in for *C. vulgaris* in literature, suggesting that dCO_2_ has little role in lipid production in such scenarios; (2) [HCO_3_^−^] change was small in the tested pH range; and (3) [CO_3_^2−^] increased quick with pH as shown in Table 2. In other words, CO_3_^2−^ was the only DIC species changed significantly in corresponding to the dramatically increase of lipid accumulation when culture pH increased from 7.5 to 9.5 or even 10.0. If this is true, the same argument could be applied to the lipid-stimulating effects of pH in NaCl cultures or in other scenarios without addition of NaHCO_3_. After all, DIC was supplied in all these scenarios and the proportion of individual species in DIC depends on pH only when temperature is constant, although the 160 mM NaHCO_3_ cultures would have contained much high [CO_3_^2−^] than in 160 mM NaCl at a given pH level. This, in fact, is compatible with the observation that 160 mM NaHCO_3_ was a much stronger lipid stimulus than the same concentration of NaCl. Nevertheless, the evidences we have are circumstantial and there are no other experimental or literature data to our knowledge to support this hypothesis at this point. Furthermore, these arguments do not exclude the possibility that the culture pH itself played an important role in lipid stimulation.

### Effects of NaHCO_3_ concentration on cell growth of *C. vulgaris*

**Fig. 4:**
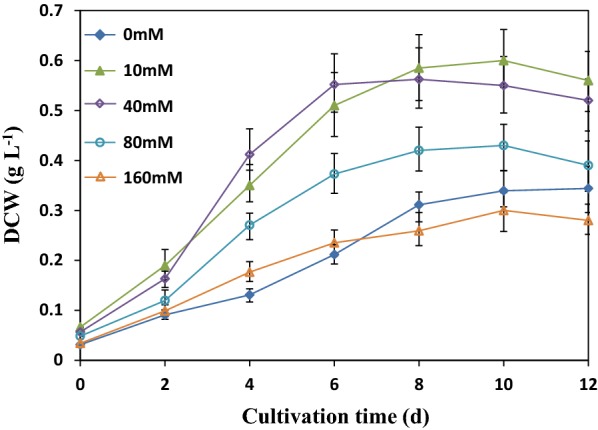
Growth of *C. vulgaris* at different concentration of NaHCO_3_ at pH 9.5. Experiments carried out in cultivation bottles. Values are the mean ± standard error of triplets

While comparison between 160 mM NaCl and 160 mM NaHCO_3_ at different pH levels offered insights on the effects of total DIC and individual DIC species on *C. vulgaris* cells, it was deemed important to compare the cell growth of *C. vulgaris* at a series of NaHCO_3_ concentration to more precisely understand the concentration effects. As shown in Fig. 4, the cell growth of *C. vulgaris* was improved by adding NaHCO_3_ in the range of 10–80 mM but the highest DCW of 0.60 g L^−1^ (which nearly doubled that of the control at 0.33 g L^−1^) was obtained in 10 mM NaHCO_3_ cultures. The benefit of low NaHCO_3_ at around 10 mM to cell growth of *C. vulgaris* is compatible with the results of Yeh et al. [35], who reported that the highest biomass concentration was obtained at 1200 mg L^−1^ (i.e. 14.3 mM) NaHCO_3_ for cultivation of *C. vulgaris* ESP-31.

The beneficial effects of low NaHCO_3_ to cell growth of *C. vulgaris* was likely due to the increase of total DIC concentration in culture, which made carbon source more abundant than in the control. The decrease of cell growth of *C. vulgaris* when NaHCO_3_ increased from 10 to 40 mM and beyond indicate that NaHCO_3_ of 40 mM or above is inhibitive to cell growth of *C. vulgaris* and the inhibitive effects increased with NaHCO_3_ concentration. Eventually, the inhibitive effects of high NaHCO_3_ overtook the beneficial effects of increased carbon source availability when NaHCO_3_ was above 80 mM, i.e., 160 mM. In summary, the results showed that the inhibition to *C. vulgaris* by DIC became significant at 40 mM and increased with NaHCO_3_ concentration from that point on. It should be pointed out that the culture pH was not controlled in the cultivation bottles and culture pH typically increased from 8.0 in the beginning to 10.5 at the end. As a result, the observed were lumped effects of DIC and pH. It is worth noting that this series of experiments was carried out under sterile conditions due to protozoa contamination concerns at low NaHCO_3_ concentrations.

## Conclusions

Comparison between cell growth in 160 mM NaCl and 160 mM NaHCO_3_ cultures at three pH levels, i.e., pH 7.5, 8.5, and 9.5, suggest that the former was beneficial while the latter inhibitive to cell growth. Results also showed that NaHCO_3_ was a stronger lipid stimulus than NaCl at these pH levels. In terms of cell morphology, 160 mM NaHCO_3_-caused *C. vulgaris* cells to form colonies after a period of cultivation. These distinctively different effects separate the effects of NaHCO_3_ from that of NaCl, or salinity.

Transformation of unicellular cells to colonies seems to be employed by *C. vulgaris* as a mechanism to mitigate the stress of high concentration of NaHCO_3_, which was confirmed by the increased shear sensitivity of *C. vulgaris* in such cultures. The colonial cells was adversely affected by a mild increase of agitation from 100 to 200 rpm in 160 mM NaHCO_3_ medium while unicellular *C. vulgaris* cells are well known for their tolerance to shear stress.

Data at different pH level with either NaHCO_3_ or NaCl indicate that increasing it in the range of pH 7.5 to pH 9.5 would increase lipid production. However, while pH increase in the same range was inhibitive to cell growth in NaCl cultures, the optimal pH for cell growth of *C. vulgaris* in NaHCO_3_ cultures shifted to pH 8.5. The inhibition of cell growth of the near-neutral pH of 7.5 in NaHCO_3_ cultures, which was beneficial to cell growth in NaCl cultures, was attributed to the high dCO_2_ under such conditions. On the other hand, the stimulus effects of DIC to lipid production is tentatively attributed to the high [CO_3_^2−^] at high culture pH.

Experiments on cell growth of *C. vulgaris* in cultures containing 0–160 mM NaHCO_3_ indicate that low NaHCO_3_ (i.e., 10–80 mM) could improve cell growth, possibly by increasing DIC concentration as additional carbon sources. Cell growth inhibition was observed starting at 40 mM and the inhibitive effects overtook the beneficial effects at 160 mM. Since this series of experiments was carried out in cultivation bottles without pH control, studies in PBR with tight pH would allow more accurate interpretation of the dependence of NaHCO_3_ effects on concentration. Such a study may also provide more accurate information on the morphological responses of *C. vulgaris* cell to the NaHCO_3_ stress.

## Abbreviations

CA: carbonic anhydrase
MBM: modified Bristol medium
PBRs: photobioreactors
LC: lipid content
LP: lipid productivity
DCW: dry cell weight
dCO_2_: dissolved CO_2_
